# Effects of three different diets on the fatty acid profile and sensory properties of fresh Pecorino cheese “Primo Sale”

**DOI:** 10.5713/ajas.19.0452

**Published:** 2020-01-13

**Authors:** Isa Fusaro, Melania Giammarco, Michael Odintsov Vaintrub, Matteo Chincarini, Anna Chiara Manetta, Ludovica M. E. Mammi, Alberto Palmonari, Andrea Formigoni, Giorgio Vignola

**Affiliations:** 1 Faculty of Veterinary Medicine, University of Teramo, Località Piano D’Accio, 64100 Teramo, Italy; 2 Department of Veterinary Medical Sciences, Alma Mater Studiorum University of Bologna, via Tolara di Sopra 50, 40064 Ozzano Emilia, Bologna, Italy

**Keywords:** Pecorino Primo Sale, Fresh Cheese, Extruded Linseed, Fatty Acids, Pasture, Sensory Properties

## Abstract

**Objective:**

This study aimed to evaluate and compare the effects of three different diets on the fatty acids (FA) profile and sensory properties of a characteristic Italian fresh cheese: Pecorino “Primo Sale” (PS).

**Methods:**

Fifty-four sheep were divided into three feeding groups: total mixed ration (TMR) enriched with extruded linseed (TL), control diet with TMR without any integration (TC), and pasture (P). During cheese production, six cheeses per experimental group were produced each week, stored for 10 days at 4°C, and then analyzed for chemical composition, FA profile, and sensory properties.

**Results:**

Saturated fatty acids (SFA) were significantly higher in PS from group TC (82.11%) than in cheese from other two groups (P 75.48% and TL 66.83%). TL and P groups presented higher values of polyunsaturated fatty acids, 4.35 and 3.65%, respectively, than that of TC group (2.31%). The lowest SFA/unsaturated fatty acid ratio was found in TL and P groups, while the highest was found in the TC group. Vaccenic acid and conjugated linoleic acid (CLA) were higher in group P (p<0.05) than in groups L and TC. Sensory properties of cheese from group P received the highest scores for odor intensity and friability, while control group had a greater chewing consistency. Overall, all cheeses received good scores for acceptability.

**Conclusion:**

In conclusion, this study showed how the integration of extruded linseed improved the FA profile of fresh pecorino cheese PS preserving its sensory properties. Levels of CLA in the PS group achieved using this approach was not higher than that in a grazing diet. Cheeses from groups P and L contained a higher value of FA, with nutritional implications for humans, when compared with un-supplemented diet. Linseed may be a good feeding strategy when pasture is not available.

## INTRODUCTION

The Mediterranean diet includes several types of fresh cheese products, such as Pecorino “Primo Sale (PS)”, which is a fresh cheese made from sheep’s milk characterized by a white color and without an evident rind. The name itself means “first salt” and is used to describe a cheese that is consumed immediately after the first salting with a short storage period (maximum 15 to 20 days) and differs from other varieties of Pecorino cheese, which usually have a long period of ripening (greater than 60 days). It is a product typical of Central and Southern Italy, where the most common dairy sheep system used to produce milk for pecorino cheese manufacture, like PS, includes native or cultivated pastures. The use of pasture has a key role in milk and dairy products, remarkably improving nutraceutical compounds, such as polyunsaturated fatty acids (PUFA) [1]. Usually, the concentration of PUFA in dairy foods can be increased with pasture or with the use of extruded linseed (EL) supplementation in the ewes’ diet [1]. EL supplementation in the diet enhances the production of the most important fatty acids (FA) for human health, such as rumenic acid (cis 9 trans11 C18:2), an isomer of conjugated linoleic acid (CLA) and PUFA n-3 (including eicosapentaenoic acid [EPA], and docosahexaenoic acid [DHA]) [2].

Studies have highlighted these FA for their effects at reducing low-density lipoprotein and cholesterol as well as for their anti-inflammatory, anti-atherogenic, and anti-carcinogenic effects in humans [2].

Therefore, producers are interested in obtaining fresh cheese with healthy characteristics throughout the year, especially when pasture is not available, as occurs during the winter or summer in Southern and Central Italy. To the best of our knowledge, no studies reported in the literature have compared the fresh pecorino PS obtained from grazing sheep with that from sheep fed indoors with a supplemented or un-supplemented diet.

The aim of this study was to evaluate and compare the FA profile and sensory properties of pecorino PS from sheep fed a diet supplemented with linseed or an un-supplemented diet (farm ration) and from grazing animals.

## MATERIALS AND METHODS

### Animals, experimental diets, and feeding routine

The experiment was performed according to Directive 2010/ 63/EU of the European Parliament (European Union, 2010) and Directive 86/609/EEC (European Economic Community, 1986), which is associated with the protection of animals used for scientific purposes. No animals were sacrificed during this study, which was carried out in accordance with the guidelines provided by the Animal Welfare Committee of the University of Teramo, Italy. The study was conducted at a farm located in the Latium region, province of Viterbo (Italy), over a period of approximately 80 days. The 80-day period was considered appropriate because oilseed supplementation in dairy animals is a two-stage process that requires at least 20 days for the diet change to exert an effect [3].

Three-weeks before the expected date of parturition, 54 Comisana ewes were randomly divided into three homogeneous groups, balanced for age (32±2 months), body weight (47.5±1.2 kg), and number of lactations (2.3±0.5). The selected ewes were randomly allotted to the following three experimental treatment groups (18 sheep per group):

- Pasture group (P): ewes had daily access to pasture for 22 h/d without supplementation (average stocking rate: 15 ewes/ha). The pasture primarily consisted of Sulla (*Hedysarum coronarium*) as well as oats (*Avena sativa*) and clover (*Trifolium incarnatum*) seeded the previous fall. This composition is typical for pastures in the Latium region.
- Control group (TC): ewes had no access to pasture but were housed in straw-bedded pens and received a winter ration as practiced in Central Italy. Ingredients of the total mix ration (TMR-C) were grass hay at 1,100 and 800 g/d of concentrate-based meal (oat, barley, and soybean) without added fats or supplements.
- Linseed-enriched group (TL): ewes had no access to pasture but were housed in straw-bedded pens and received a winter ration as practiced in Central Italy with addition of extruded linseed. The ingredients of the TMR-L were grass hay at 1,100 and 800 g/d of concentrate-based meal with the addition of 0.190 kg of extruded linseed. Linseed, ground to pass through a 4-mm screen, was extruded in a single screw extruder with a throughput of 1,600 kg/h (barrel length: 3.2 m; die diameter: 7 mm; screw speed: 300 rpm; temperature at the end of the barrel: 130°C to 138°C; duration: 1 min). After extrusion, the product was dried in a counter flow cooler for 12 min. The ingredients and the chemical compositions of the three diets are showed in Table 1.

### Primo Sale sampling

Following the removal of lambs at 4 weeks of age, the ewes were machine-milked twice a day at 6 am and 6 pm. From weeks 5 to 8, milk was collected daily from ewes in each treatment group and refrigerated for pecorino production three times a week. Following each cheese-making process, six pieces of cheese, about 1 kg each, were taken from each experimental group per week, stored for 10 days at 4°C, and then analyzed.

Briefly, bulk milk was pasteurized at 72°C for 20 s, cooled to the coagulation temperature (37°C to 39°C), and then transferred to a container with 500 UI/5,000 L of combined thermophilic and mesophilic starter cultures (WhiteDaily, Chr Hansen, Hoersholm, Denmark). After acidification, rennet (15 g/100 kg; 75% of chymosin, and 25% of pepsin; 1:18,000 strength; Clerici, Cadorago, Italy) was added to the milk; coagulation began after 30 min of incubation. The curd was broken into small pieces the size of hazelnuts, portioned in aliquots of 1 kg, transferred into plastic molds, and kept at 48°C±1.5°C until the pH reached 5.20±0.1. Then, a 20% NaCl water solution was used to salt the cheese in brine. Thereafter, the salted fresh cheese was stored at 4°C ±0.5°C.

### Chemical analysis of the diets and Primo Sale composition

During the trial, samples of pasture and TMR were collected and analyzed for dry matter (DM) content, crude protein (CP), and ether extract (EE), following the official methods of the AOAC 1990 (Association of Official Analytical Chemist) [4]. Neutral detergent fibre (NDF), acid detergent fibre (ADF), and acid detergent lignin (ADL) were analyzed as described by Van Soest et al [5]. DM, total proteins, and ash levels were determined according to AOAC International for the cheeses [6].

Cheese lipids were extracted by acid hydrolysis with 20 mL of ethanol and 500 μL of hydrochloric acid 3 N. fatty acids methyl esters (FAME) were extracted using a mix of chloroform and methanol (2:1, vol/vol). Methylation included 70 mg of lipids reconstituted with 1 mL of hexane and 500 μL of sodium methoxide in methanol (1:1, vol/vol). Separation of FAME was performed by gas cromatatography (GC) using a GC (Thermo Scientific, Waltham, MA, USA) equipped with a capillary column (Restek Rt-2560 Column fused silica 100 m×0.25 mm highly polar phase; Restek Corporation, 108 Bellefonte, PA, USA) and a flame ionization detector (FID). The initial oven temperature was 55°C, which was held for 4 min and subsequently increased to 175°C at a rate of 13°C/min, where it was held for 27 min, increased to 215°C at a rate of 4°C/min, and then held for 45 min. Hydrogen was used as the carrier gas and the column head pressure was 175 kPa. Fatty acids were identified by comparing their retention times with the fatty acid methyl standards (FIM-FAME-7-Mix, Matreya LLC, Pleasant Gap, PA, USA), and C18:1 trans-11, C18:2 cis-9,trans-11, C18:2 cis-9,cis-11, C18:2 trans-9, trans-11, and C18:2 trans-10, cis-12 (Matreya LLC, USA), and peak areas were quantified using Agilent Chemstation software (Thermo Scientific, Waltham, MA, USA).

The surface color of cheeses was measured using a Minolta Chromometer CR-300 (Minolta, Osaka, Japan) with CIELAB L*a*b* values [7]. The measure of lightness (L* values, range 0 to 100) represents black to white, the measure of redness (a* values) describes green to red, and the measure of yellowness (b* values) represents blue to yellow.

Color measurements were made on fresh cut surfaces and represent the mean of three measurements performed on the cross-section of the cheese.

### Evaluation of lipid oxidation in cheese by thio-barbituric acid reactive substances-test

Thio-barbituric acid reactive substances (TBARS) were measured using a method described by Kristensen and Skibsted [8] to determine lipid oxidation in cheese. An aliquot of 6.0 g of cheese, exactly weighted, was taken from each sample, 18.00 mL of previously prepared TBA reagent was added, and the resulting mixture was homogenized using an Ultra Turrax (Heidolph, Diax 600, Staufen, Germany) for 2 min until the mixture appeared to be homogeneous. An aliquot (6 mL) of the suspension was transferred to a Pyrex tube, mixed with 3.5 mL of chloroform, and then mixed gently for 5 min.

Subsequently, the mixture was centrifuged at 6,000×*g* for 15 min. The aqueous solution was placed in a water bath at 100°C for 10 min and then cooled on ice. The orange-red cyclohexan supernatant was decanted and the absorbance at 532 nm was measured by spectrophotometry (JENWAY 6305UV/Vis, Jenway, Essex, UK). The results were expressed as absorbance units at 532 nm per gram of cheese.

### Primo Sale sensory properties and acceptability test

The sensory properties of cheese were analyzed using a previously described method, with adaptations [9]. The assessment was performed by a sensory panel composed of 15 panelists, trained to follow the criteria of ISO 8586-1:1993 standards [10]. The panelists were not provided with any information regarding the samples to be tasted. In the test, cheese cube samples (1 cm^3^) were served in random order in a sensory analysis laboratory at room temperature (18°C±1°C). A 0 to 9 scale was used for to quantify each sensory attribute, with 0 referring to the minimum intensity and 9 to the maximum intensity, according to sensory analysis methodology [11]. The attributes were: color, paste homogeneity, greasiness, odor intensity, foreign aromatic smells, chewing consistency, friability, saltiness, acidity, bitterness, sweetness, foreign tastes, and overall acceptability.

Primo Sale cheeses were also evaluated for consumer acceptability [12]. A total of 100 untrained consumers aged 35 to 45 years and balanced for sex participated in the test. Cubed (1 cm^3^) cheese samples were coded with three-digit random numbers and served in a sensory analysis laboratory at room temperature (18°C±1°C). Each panelist was also provided with unsalted crackers and a glass of water. For each product, consumers expressed their overall liking and dislike according to the following sensory inputs: appearance, taste/flavor, and texture. Consumers rated their liking on a nine-point hedonic scale labeled on the left end with “extremely unpleasant” [1], at the right end with “extremely pleasant” [9], and in the center with “neither pleasant nor unpleasant” [10].

### Statistical analysis

Prior to statistical analysis the data on FA composition were processed and the following FA classes and indices were calculated: monounsaturated fatty acid (MUFA, all FAs with a single double bond), PUFA (all FAs with more than one double bond); saturated fatty acids (SFA, all FAs without double bonds); unsaturated fatty acid (UFA, all FAs with one or more double bonds); n-3 (ΣC18:2 t11, c15 + C18:2 c9, c15 + C18:3 c9, c12,c15 + C:22:5 c7, c10, c13, c16, c19 + EPA + DHA); n-6 (ΣC18:2t9, t12 + C18:2 c9, t12 + C18:2 t9, c12 + C18:2 c9, c12 + CLA t10,c12 + C20:2 c11, c14 + C20:3 c8, c11, c14 + C20:4 c5, c8, c11, c14); the atherogenic index (AI) was calculated according to Ulbricht and Southgate [13], as follows: (C12:0 + 4×C14:0 + C16:0)/(monounsaturated + PUFAs); I-Harris index as a sum of EPA and DHA [14].

All data on the chemical and fatty acid composition of cheese were analyzed using general linear model repeated measures of the SPSS version 13.0 statistical package, including the fixed effects of dietary treatment and sampling time. The effect of cheese making time is not reported in the tables because it was not significant. The significance of the fixed effects is presented for each parameter evaluated and the variance is expressed as standard error. When the variance analysis was significant (p<0.05) the differences between the means were compared by the least significant difference test.

Sensory and acceptability data were normalized and subjected to analysis of variance for repeated measures with diet as the sole factor. Duncan’s test was used to determine the whether the groups were significantly different from each other.

## RESULTS

### Chemical composition of Primo Sale

The chemical composition of PS is shown in Table 2. The percentage of CP differed between the three groups at 37.59% (TL), 40.86% (TC), and 45.05% (P). The total fat percentage of the PS was affected (p<0.05) by dietary treatment; cheeses made from milk of the TL and TC groups had a higher fat content (47.30% and 48.41%, respectively) than cheese from the P group (44.32%). There was no significant difference in the DM of the cheeses between the groups, at 57.62%, 59.64%, and 57.32%, in the TC, TL, and P groups, respectively.

The TBARS, pH, water activity (aw), and acidity of PS were not influenced by dietary treatment. Conversely cheeses from the TL group presented higher luminosity (L*) than those from the TC and P groups, while cheese from the P group presented higher levels of response on the yellow-blue scale (b*). A negative value on the red-green scale (a*) was recorded for all three groups, with no significant difference.

### Fatty acid profile of Primo Sale

The FA analysis of PS is shown in Table 3. The proportion of total SFA was higher in PS (p<0.05) from the TC group (82.11%) compared with the products obtained from sheep-fed pasture (75.48%) or extruded linseed diets (66.83%). A significantly higher concentration of PUFA (p<0.001) was observed in cheese from the TL group (4.35%) compared with cheese from the TC group (2.31%), whereas an intermediate value was observed with P treatment (3.55%). Overall, the higher ratio of SFA/UFA was found in the TL and P groups, which were very similar (2.02% and 2.58%, respectively), whilst the lowest was found in the TC group (3.20%). The content of n-3 FA was significantly higher (p<0.001) in PS from the TL group than in PS from the TC and P groups; in the latter group, the n-3 content was at an intermediate level.

Significantly higher levels (p<0.05) of vaccenic acid (VA) and CLA were observed in the PS from group P compared to that from group TC, while the values of VA and CLA from group TL were higher than those from TC but lower than those from P (p<0.05). The I-Harris index, which is the sum of EPA+DHA, was significantly higher in PS from the P and TL groups than in PS from the TC group (p<0.05). The AI was lower (p<0.001) in the L group than in the TC group, whereas the value in the P group was slightly higher than that in the TL group.

### Sensory properties of Primo Sale

The sensory properties of cheese are shown in Table 4. Extruded linseed supplementation and pasture diet affected the color and paste homogeneity (p<0.05). Color homogeneity was higher (p<0.05) in groups P and TL than in group TC, while paste homogeneity was lower (p<0.05) in group TC than in groups P and TL. Higher scores (p<0.05) in cheese of group TC compared to that from groups P and TL were registered for chewing consistency. Friability was significantly (p<0.05) higher in groups P and TL. PS from group P also scored higher for odor intensity (p<0.05). All cheeses received good scores for their overall acceptability, with no statistically significant differences in the mean values between groups P and TL, which were 6.38 for TL and 6.48 for P.

## DISCUSSION

Pasture influenced the CP content of cheese (p<0.05), and this effect could be due to the different composition of the diet. The content of NDF and ADL are lower in pasture; therefore, our hypothesis is that the diet with pasture resulted in higher digestibility, which can also influence the total DM intake and the nutrient availability for rumen microbiota and consequently for animals. The EE of cheeses was due to lower levels of NDF in the P diet, which can reduce acetate production in the rumen. As expected, the tatty acid composition in pasture is characterized by the highest content of linolenic acid, which can influence the bio-hydrogenation process in the rumen leading the production of some intermediates (C18:2 trans 10, cis 12) that can depress the *de novo* synthesis of fatty acids in the mammary gland. These results are consistent with previous results in sheep and cows [15,16]. This response has been previously reported in dairy sheep fed fresh Italian ryegrass with a low level of NDF, as in the present study [17].

There was no difference in the TBARS value between the three groups of cheese. These results may be due to the freshness of PS (10 days of aging), with a low level of lipid oxidation, which normally occurs after long periods of seasoning [18]. The literature report that a high concentration of PUFA and a long ripening time predispose the cheese to a greater lipid oxidation [19]. These results indicated that in a fresh cheese with a high PUFA concentration, such as those of groups P and TL, the oxidation level does not different compared to that in cheese with poor PUFA, such as the cheese from group TC.

Instrumental color analysis revealed several differences between three groups. The b* values were significantly higher in PS from the P group compared with that from the other groups, which was not surprising due to the high levels of carotenoids present in the pasture-based diet. Similar results were found in cheese from grazing cows [20]. Lightness values (L*) of linseed-enriched products have provided contrasting results in other studies. In our study, color differences among groups could be due to the higher concentration of SFA in unsupplemented cheeses than in those from groups P and TL. Hurtoud and collaborators [21] reported no significant effects of linseed supplementation on the color analysis of butter samples, while Lerch et al [22] observed higher L values in milk products from cows supplemented with EL. The level of EL integration in the diet could influence the color parameters. Based on our experience, we suggest that integration of 0.190 kg/capo/die could affect the instrumental color of PS.

There were significant differs in FA, which has nutritional implications for human health (Table 3). The significantly lower SFA content in group TL compared to that in groups P and TC indicate a healthier cheese product. A meta-analysis investigated the association between milk and dairy consumption and cardiovascular diseases (CVD), and reported that high levels of SFA in dairy products are associated with a high risk of CVD [2]. Linseed supplementation and pasture resulted in a lower SFA/UFA ratio compared to the TC group, due to the lower concentration of SFA in P and TL, and the higher value of PUFA and MUFA compared to the TC group. These results are consistent with those of Cabiddu et al [17]. The scientific literature state that dairy products enriched with n-3FA are considered healthy. Therefore, the levels of these beneficial fatty acids are fundamental in the prevention of CVD; however, unfortunately, in milk products the concentrations of EPA and DHA are highly variable compared to fish and vegetable oils [10]. Our results revealed a higher value for I-Harris index (the sum of EPA and DHA) in P and TL groups compared to the TC group; EPA and DHA represent the major n-3 PUFAs, which play key physiological roles in health and disease. These two FAs are the elongation-desaturation products of n-3 FAs, such as alfa linoleic acid (ALA); in human tissue only 5% to 15% of ALA could be converted in EPA and less than 1% of ALA is reliably converted to DHA [23]. ALA is a major component of n-3 FAs; therefore, it is important to obtain dairy products with high n-3FAs concentration. Cabiddu et al [17] found a better n-3 FA profile in milk from fresh pasture, whilst our results showed that the n-3FA content was higher in cheese from the TL group compared to that from the P group. This is probably due to a different kind of pasture, which in our study, was a mixture of legumes and sulla while in that of Cabiddu et al [17] was the Italian ryegrass (*Lolium mutiflorum*), underlining the better ALA/linoleic acids (LNA) ratio in pasture compared to the supplemented groups.

The higher value of C18:1 trans-11 (VA) in PS from groups P and TL compared with that from TC was not surprising, because specific C18 chains are found at higher concentrations in these diets. VA is a common intermediate in the biohydrogenation of both linoleic and linolenic acids, and the concentration of VA in milk is directly related to the diet [24]. The higher concentration of VA in group P than in groups TC and TL may be due to the increased levels of *Butyrivibrio fibrisolvens* (*B. fibrisolvens*) in the rumen of sheep fed with pasture. *B. fibrisolvens* is responsible for the hydrogenation of LNA and linolenic to VA [25]. In our studies, it is possible that LNA contained in extruded linseed was not easily accessible to ruminal microbiota or encumbered the action of *B. fibrisolvens* differently than did pasture [25]. However, pasture may not be a stable source of FA, and cheese FA quality tends to vary during the year, following seasonal grazing quality and availability. Among the cheese obtained from barn-held ewes, the PS of group TL had a higher content of VA, which is directly associated with the positive effect of linseed on C:18 FA isomers in the milk of dairy animals. Similarly, the levels of rumenic acid, the principal isomer of CLA, were significantly higher in PS from group P (p<0.05) than in PS from group TC. These results demonstrate that the use of linseed or pasture can improve the concentration of CLA in fresh pecorino cheese PS better than an unsupplemented diet.

Most previous studies on the sensory properties of Pecorino were conducted on ripening pecorino cheese. This was the first study to investigate fresh pecorino cheese obtained with different feeding regimes and the effects of animal diets on cheese sensory properties. Cheese from groups TC and TL received lower color scores. This may be due to the higher concentration of carotenoids in pasture. Carotene is a pigment present in large amounts in green forage, which contributes to the yellow coloration of dairy products [26]. The higher yellowness intensity perceived by panelists for P cheeses confirmed the results concerning instrumental color (Table 2), with a higher b* index reported for the same cheeses. The high score for chewing consistency of cheeses from group TC could be attributed to the higher content of SFA, which gives the cheese a more solid structure. Caroprese and co-workers [27] found that PUFA- and CLA-enriched butter and cheese were softer and less firm than conventional butter and cheese. The higher friability values of cheeses from groups P and TL could also be correlated to the less rigid structure due to the higher PUFA content, making it less difficulty to fracture compared to that of the TC group. According to Esposito et al [28], the pasture-based diet resulted in a less difficulty to fracture, moreover, it received a higher score for floral and herbaceous odors. Similar results were obtained by Bonanno et al [29]. The odor intensity was significantly higher in cheese from group P, with no significant differences observed between groups TC and TL. The impact of pasture on odor intensity has been documented for other cheese products [30]. Overall, all cheeses received positive evaluations for acceptability, even if untrained consumers were not able to detect the differences that could be discriminated by the trained panelists between PS cheeses.

## CONCLUSION

This work highlighted how the use of extruded linseed influenced the fatty acid composition of fresh pecorino cheese “Primo Sale”. The most interesting aspect of the present study was the comparison of fatty acid profiles of cheese obtained from ewes fed an extruded linseed and pasture diet. In fact, extruded linseed supplementation was more effective at reducing the SFA and increasing the PUFA n-3 FA in cheese compared to that obtained from pasture ewes, indicating that consumption of these products may have a positive effect on human health.

However, levels of CLA in PS achieved using this approach were not higher than that in a grazing diet. This study demonstrated that the integration of sheep diets with linseed improved the fatty acid profile of fresh pecorino cheese “Primo Sale” and could be a good feeding strategy to apply in ewe farms when pasture is not available.

Nevertheless, pasture is a viable option relative to an unsupplemented diet because it combines a good product quality with low feeding costs. The cheese obtained from grazing animals received a high score for color and odor perception by trained panelists and received a good score for acceptability as the PS cheeses obtained from other two treatment groups.

## Figures and Tables

**Table 1:** Ingredients (% DM), chemical composition and fatty acid composition of the experimental diets

Items	Dietary treatment1)		

	TC	TL	P
Ingredient			
Grass hay	56.79	56.79	-
Oatmeal	12.91	10.33	-
Barley meal	12.91	10.33	-
Soybean meal	15.85	11.20	-
Extruded linseed	-	9.81	-
Mineral and vitamin mix	1.55	1.55	-
Chemical composition			
DM	89.12	90.56	16.72
CP	20.71	19.97	19.57
EE	2.86	4.83	2.50
NDF	39.24	44.75	29.77
ADF	25.48	25.2	21.91
ADL	3.68	3.93	1.41
Fatty acid profile (% of total FA)			
C12:0	0.15	0.18	0.17
C14:0	0.6	0.2	0.30
C16:0	18.8	8.8	12.7
C16:1	1.01	0.98	1.1
C16:1c9	0.3	0.1	0.2
C18:0	2.6	4.1	1.11
C18:1c9	23.8	21.1	1.81
C18:2c9, c12	40.0	13.3	11.5
C18:3c9, c12, c15	9.1	42.9	71.2
C18:1c11	1.0	0.6	0.8

DM, dry matter; FA, fatty acid; CP, crude protein; EE, ether extract; NDF, neutral detergent fiber; ADF, acid detergent fiber; ADL, acid detergent lignin.

1) TC, unsupplemented diet; TL, diet supplemented with extruded linseed; P, pasture based diet.

**Table 2:** Chemical composition (% of DM), thiobarbituric acid reactive substances, pH, and color coordinates (L*, a*, b*) of Primo Sale cheese from ewes fed with three different diets

Items	Dietary treatment1)			Significance2)	SE

	TC	TL	P		
DM	57.62	59.46	57.32	NS	1.23
Ash	7.12	6.99	6.15	NS	1.89
CP	40.86^b^	37.59^a^	45.05^c^	*	1.25
EE	48.41^b^	47.30^b^	44.32^a^	*	1.89
TBARS	0.47	0.34	0.38	NS	1.65
pH	5.19	5.18	5.20	NS	1.74
L*	76.41^a^	81.97^c^	79.92^b^	*	0.98
a*	−2.45	−2.31	−2.11	NS	0.32
b*	13.30^a^	13.84^a^	14.67^b^	*	0.21

SE, standard error; DM, dry matter; CP, crude protein; EE, ether extract; TBARS, thiobarbituric acid reactive substances.

1) TC, unsupplemented diet; TL, diet supplemented with extruded linseed; P, pasture based diet.

2) NS, not significant; * p<0.05.

a–c Different letters in the same row indicate significant differences (p<0.05).

**Table 3:** Content of some classes of fatty acids (expressed in mg/100 g) and indices of Primo Sale made from ewes fed with three different diets

Items	Dietary treatment1)			Significance2)	SE

	TC	TL	P		
SFA	82.11^c^	66.83^a^	75.48^b^	**	1.64
MUFA	15.51^A^	28.64^B^	25.60^B^	***	1.90
PUFA	2.31^a^	4.35^c^	3.55^b^	*	0.54
UFA	17.82^a^	32.98^c^	29.15^b^	*	1.11
SFA/UFA	4.60^b^	2.02^a^	2.58^a^	**	1.58
n-33)	0.48^c^	1.79^a^	0.78^b^	**	0.69
n-64)	1.46	1.37	1.31	NS	1.35
n-3/n-6	0.33^a^	0.91^c^	0.59^b^	*	1.56
CLA	0.31^a^	0.56^b^	0.77^c^	*	2.01
VA	1.02^a^	2.25^b^	2.99^c^	*	0.98
AI5)	5.28^C^	1.89^A^	3.05^B^	***	1.07
I-Harris6)	0.02^a^	0.09^b^	0.08^b^	*	1.87

The data correspond to analysis of fresh Primo Sale cheese from bulk tank milk.

SE, standard error; SFA, saturated fatty acid; MUFA, monounsaturated fatty acid; PUFA, polyunsaturated fatty acid; UFA, unsaturated fatty acid; CLA, conjugated linoleic acid; VA, vaccenic acid (C18:1 trans −11); AI, atherogenic index; EPA, eicosapentaenoic acid; DHA, docosahexaenoic acid.

1) TC, un-supplemented diet; TL, diet supplemented with extruded linseed; P, pasture based diet.

2) * p<0.05; ** p<0.01; *** p<0.001; NS, not significant.

3) n-3 = (C18:2 t11, c15 + C18:2 c9, c15 + C18:3 c9, c12,c15 + C:22:5 c7, c10, c13, c16, c19 + EPA + DHA).

4) n-6 = (C18:2t9, t12 + C18:2 c9, t12 + C18:2 t9, c12 + C18:2 c9, c12 + CLA t10,c12 + C20:2 c11, c14 + C20:3 c8, c11, c14 + C20:4 c5, c8, c11, c14).

5) AI = (C12:0 + 4 × C14:0 + C16:0)/(MUFA + PUFA).

6) I-Harris = (EPA + DHA).

Different letters in the same row indicate significant differences (^a–c^ p<0.05, p<0.01; ^A–C^ p<0.001).

**Table 4:** Sensory properties ratings (scale 0 to 9) and acceptability test of Primo Sale made from ewes fed with three different diets

Items	Dietary treatment1)			Significance2)	SE

	TC	TL	P		
Colour	6.04^a^	6.45^a^	7.64^b^	*	0.92
Paste homogeneity	5.96^a^	6.57^b^	6.22^b^	*	0.56
Greasiness	6.50	6.31	6.33	NS	0.25
Odour intensity	5.32^a^	5.34^a^	6.69^b^	*	0.98
Foreign aromatic smells	1.32	1.38	1.26	NS	0.58
Chewing consistency	5.36^b^	4.34^a^	4.56^a^	*	0.78
Friability	4.43^a^	5.86^b^	5.52^b^	*	0.56
Saltiness	5.32	5.83	5.33	NS	0.15
Acidity	1.79	1.55	1.74	NS	0.14
Bitterness	3.57	3.41	3.22	NS	0.89
Sweetness	4.64	4.72	4.15	NS	0.56
Foreign tastes	1.29	1.48	1.19	NS	1.01
Acceptability test					
Overall liking	7.64	7.83	7.48	NS	0.89
Appearance	6.52	6.89	6.24	NS	0.77
Taste/Flavour	6.12	6.35	6.25	NS	0.70
Texture	7.01	6.97	7.03	NS	0.52

SE, standard error.

1) TC, un-supplemented diet; TL, diet supplemented with extruded linseed; P, pasture-based diet.

2) * p<0.05; NS, not significant.

Different letters in the same row indicate significant differences (^a,b^ p<0.05).
